# Host-Directed FDA-Approved Drugs with Antiviral Activity against SARS-CoV-2 Identified by Hierarchical In Silico/In Vitro Screening Methods

**DOI:** 10.3390/ph14040332

**Published:** 2021-04-06

**Authors:** Tiziana Ginex, Urtzi Garaigorta, David Ramírez, Victoria Castro, Vanesa Nozal, Inés Maestro, Javier García-Cárceles, Nuria E. Campillo, Ana Martinez, Pablo Gastaminza, Carmen Gil

**Affiliations:** 1 Centro de Investigaciones Biológicas Margarita Salas-CSIC, Ramiro de Maeztu 9, 28040 Madrid, Spain; tiziana.ginex@cib.csic.es (T.G.); vanesanozal@cib.csic.es (V.N.); ines.maestro@cib.csic.es (I.M.); javier.garcia.carceles@cib.csic.es (J.G.-C.); nuria.campillo@csic.es (N.E.C.); ana.martinez@csic.es (A.M.); 2 Centro Nacional de Biotecnología-CSIC, Calle Darwin 3, 28049 Madrid, Spain; ugaraigorta@cnb.csic.es (U.G.); vcastro@cnb.csic.es (V.C.); 3 Instituto de Ciencias Biomédicas, Universidad Autónoma de Chile, Llano Subercaseaux 2801—piso 6, Santiago 7500912, Chile; david.ramirez@uautonoma.cl

**Keywords:** SARS-CoV-2 evaluation, COVID-19, drug repurposing, host-based targets, virtual screening, entry inhibitors

## Abstract

The unprecedent situation generated by the COVID-19 global emergency has prompted us to actively work to fight against this pandemic by searching for repurposable agents among FDA approved drugs to shed light into immediate opportunities for the treatment of COVID-19 patients. In the attempt to proceed toward a proper rationalization of the search for new antivirals among approved drugs, we carried out a hierarchical in silico/in vitro protocol which successfully combines virtual and biological screening to speed up the identification of host-directed therapies against COVID-19 in an effective way. To this end a multi-target virtual screening approach focused on host-based targets related to viral entry, followed by the experimental evaluation of the antiviral activity of selected compounds, has been carried out. As a result, five different potentially repurposable drugs interfering with viral entry—cepharantine, clofazimine, metergoline, imatinib and efloxate—have been identified.

## 1. Introduction

Together with severe acute respiratory syndrome coronavirus (SARS-CoV) and Middle East respiratory syndrome coronavirus (MERS-CoV), SARS-CoV-2 is the third pathogenic and transmissible coronavirus that has emerged in humans. This new coronavirus (CoV) is the causative agent of the present pandemic of the coronavirus disease named COVID-19, first reported in Wuhan (China) [1]. Since there is no effective treatment available, and given the urgency of the pandemic, the repurposing of approved drugs is the only alternative to find a cure for the current emergency. In fact, several clinical trials are currently ongoing to prove the efficacy of old drugs in COVID-19 patients [2]. Such is the case of the drugs including in the SOLIDARITY clinical trial (remdesivir, hydroxychloroquine, lopinavir/ritonavir and interferon-beta1a), launched by the WHO in dozens of countries that showed little or no effects on hospitalized COVID-19 patients at proposed dose regimens [3]. Moreover, the only drug approved by the FDA for the treatment of extremely ill patients is remdesivir [4], an antiviral originally developed for Ebola virus infection [5].

Although in principle not very innovative, drug repurposing is a promising approach to accelerate the drug discovery process, which allows for an increase of the productivity of the pharmaceutical companies [6], and fills the gap existing in unmet diseases such as rare or infectious diseases [7,8]. In viral infections lacking effective treatment, drug repurposing combined with drug validation in animal models has enhanced the number of potential antivirals with known mechanism of action [9]. 

The COVID-19 global emergency has generated an unprecedent situation, which prompted scientists all around the world to actively work in all imaginable aspects related to SARS-CoV-2. In only a few months, the knowledge of SARS-CoV-2 significantly increased and the collection of available information today is quite large. Together with the efforts to better understand the epidemiology, virus structure and life cycle, several therapeutic targets to guide the drug discovery research have also emerged [10]. In this regard, it is remarkable the number of drug repurposing efforts trying to shed light into COVID-19 patient treatment [11,12]. Today, far from initial opportunistic and mainly serendipitous discoveries in the drug repurposing field, a number of candidates have been proposed to be repurposed for COVID-19 based on different in silico and in vitro studies [13]. 

In the attempt to proceed toward a proper rationalization of the search for new antivirals among approved drugs, we here provide a hierarchical in silico/in vitro protocol, which successfully combines virtual and biological screening to speed up the identification of anti-SARS-CoV-2 agents in an effective way.

Moreover, as viral mutations represent one of the main challenges to overcome with antiviral therapies, we carried out a multi-target virtual screening protocol focused on druggable targets related to viral entry, followed by biological screening against SARS-CoV-2 to identify host-directed therapies against COVID-19. In this regard, eight proteins mainly involved in SARS-CoV-2 entry and trafficking were considered. 

Spike glycoprotein represents a crucial factor for virus entry and thus for virus tropism, virulence and pathogenesis [14,15]. For both SARS-CoV and SARS-CoV-2, cell-virus membrane fusion is promoted by the recognition of specific host proteins, or cell-binding agents such as the angiotensin-converting enzyme 2 (ACE2), which binds the receptor binding domain (RBD) located at the S1 subunit of the head region of the protein [15]. S priming is essential to promote membrane fusion. This process is catalyzed by specific host soluble proteases as the transmembrane serine protease 2 (TMPRSS2) mainly expressed in the surface of the airway epithelial cells [16]. TMPRSS2 was demonstrated to also cleave ACE2 [17,18], enhancing viral infectivity. Proteolytic cleavage of S is also promoted by other host proteases such as furin, which have cumulative effects of TMPRSS2-mediated S priming and SARS-CoV-2 entry [19,20]. Cathepsin L, a lysosomal cysteine protease of the papain family, is also involved in SARS-CoV and SARS-CoV-2 S priming [21]. All of these findings highlight the pivotal role exerted by host proteases in viral infection [22,23], thus confirming their inhibition as a valuable strategy to tackle COVID-19.

Furthermore, the adaptor-associated kinase 1 (AAK1) and the cyclin G-associated kinase (GAK), members of the numb-associated kinase family (NAK), represent two other interesting drug targets against SARS-CoV-2 [24,25]. The main endosomal phosphatidylinositol-3-phosphate/phosphatidylinositol 5-kinase (PIKfyve) was also proposed to be related with intracellular trafficking of Ebola and SARS-CoV-2 viral particles [26]. 

Finally, the type 2 endo-lysosomal two-pore channel (TPC2), mainly expressed in late endosomes/lysosomes, mediates intracellular trafficking of coronavirus through the endo-lysosomal system. Accordingly, activation of TPC2 induces a calcium-dependent depolarization of the endo-lysosomal membrane, which is supposed to enhance S-driven membrane fusion [27]. In this context, TPC2 inhibitors such as verapamil [28], would be able to negatively affect depolarization, thus reducing the fusogenic propensity during virus-host membrane fusion.

Considering this background, the US Drug Collection of 1789 compounds of FDA-approved drugs was then virtually screened towards the eight above mentioned targets and a total of 173 FDA repurposable drugs were selected from virtual screening and subsequently experimentally evaluated. Selection of these targets was motivated by their relevant role in virus life cycle, especially in virus recognition, entry and trafficking. Primary hits were validated using viral antigen detection in infected cells. Confirmed candidates were subsequently tested for their ability to interfere selectively with viral entry in a surrogate model of infection. This process led to the identification of cepharantine, clofazimine, metergoline, imatinib and efloxate as selective SARS-CoV-2 entry inhibitors, together with a panel of non-selective entry inhibitors that could be considered also for drug repurposing to treat COVID-19.

## 2. Results

### 2.1. Virtual Screening against Selected Targets

**Figure 1:**
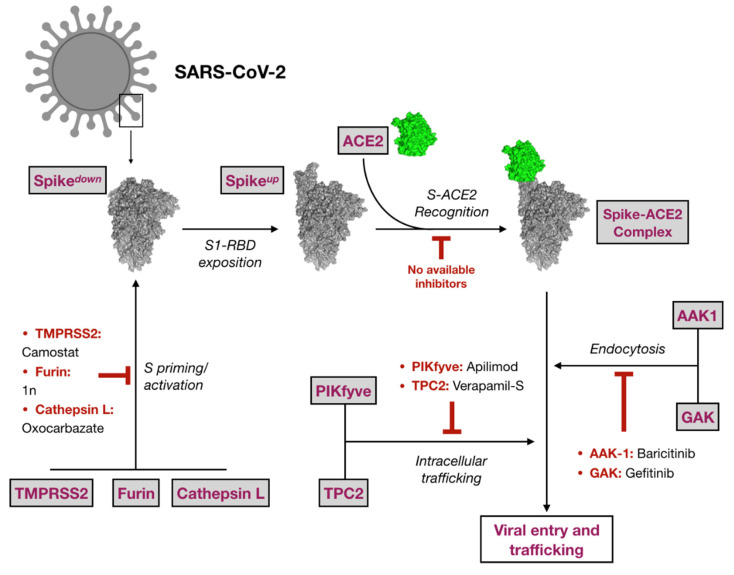
Schematic representation of the eight targets selected in this study and their role in virus entry. Representative inhibitors are also cited, when available.

A hierarchical virtual screening (VS) approach was applied on crucial SARS-CoV-2 protein targets in the attempt to find repurposable agents from the original list of FDA approved drugs. Among all the proposed druggable targets for SARS-CoV-2, eight proteins responsible for virus entry and trafficking were selected in this study. A schematic representation of their role in virus entry and trafficking is displayed in Figure 1. 

**Figure 2:**
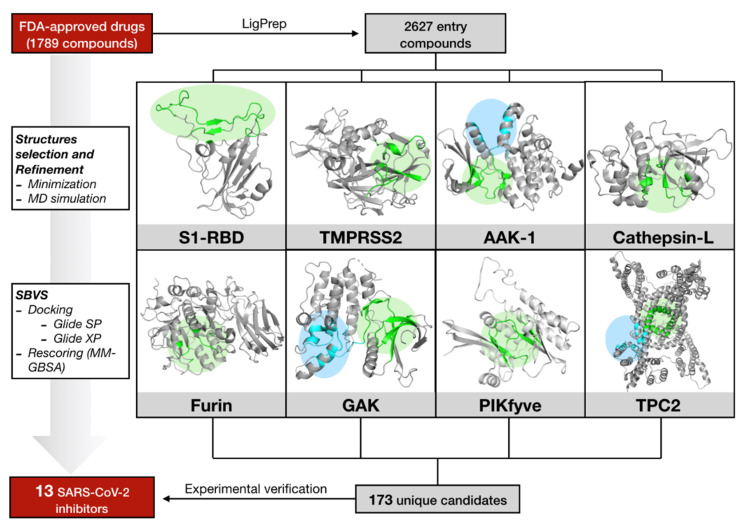
Schematic representation of the computational protocol applied in this study. For each target, green and blue circles respectively mark the active and the allosteric/secondary binding sites.

A list of 1789 FDA-approved drugs was screened on all the previously cited targets (see Materials and Methods section for the computational details) to find effective antiviral compound candidates acting on SARS-CoV-2. Details about all the available PDB structures, druggable sites explored during VS and known inhibitors are reported in Table S1 of the Supplementary Materials. The computational protocol applied in this study is shown in Figure 2. 

According to the scheme reported in Figure 2, all the systems were subjected to structural refinement by mean of energy minimization, and the minimized structures were then used for VS. A special refinement was reserved to the S1-RBD domain of Spike and to the homology modelled structured of PIKfyve and TMPRSS2. 

For PIKfyve enzyme, minimization of the homology modelled protein was realized in the presence of ATP substrate. This allowed to correctly reorient side chains for residues pertaining to the ATP binding site, preserving the geometry and shape of the cavity. 

In the case of S1-RBD and TMPRSS2, a further treatment based on molecular dynamic (MD) simulation in the NVT ensemble was applied. This allowed us to properly explore local conformational flexibility of the ACE2 binding domain of S1-RBD and to refine the homology modelled structure of TMPRSS2 prior to virtual screening. 

Trajectory analysis for S1-RBD and TMPRSS2 revealed good stability during the MD simulation (Figure S1 of the Supplementary Materials). For S1-RBD, a close analysis of the residues constituting the ACE2 recognition motif on the receptor binding region, revealed a mayor degree of fluctuation at the loop containing F154, N155 and Y157 (in dark blue in Figure S1A). Less mobility, generally lower than 1 Å, was observed in the other regions. For TMPRSS2 (Figure S1B), significant fluctuations were observed around the catalytic residues, H296, D345 and S441 (especially for loops in light blue, orange and green), which would be ascribable to the significant solvent exposition of the active site. For these two targets, the minimized structure and the most representative clusters (Tables S1 and S2 of the Supplementary Materials) obtained from MD simulations were thus used for multi-conformation VS. 

Accordingly, a total of 6 conformations for S-RBD of the Spike glycoprotein and 4 conformations for TMPRSS2 were considered for the following virtual screening (see Material and Methods section for additional information about clusters selection). For all the other targets, only the energy minimized crystallographic structure was considered (Tables S1 and S2 of the Supplementary Materials). 

All targets were subjected to a three-staged virtual screening protocol consisting on a preliminary docking by using the SP Glide docking algorithm, a second docking by applying the XP Glide docking algorithms and a final rescoring by applying the Prime MM-GBSA method. For each screened target, at least the 50 best ranked FDA drugs according to the MM-GBSA score were preliminarily selected (Table S3 of the Supplementary Materials). Among them, compounds intended for a veterinary and/or cosmetic use, biocides, laxative or topical-administered drugs were not considered for SARS-CoV-2 in vitro assays. The complete list for the 173 selected drugs and their potential target(s) emerged from VS is shown in Table S4 of the Supplementary Materials. These compounds were experimentally assayed for their SARS-CoV-2 antiviral potential within the framework of a host-directed COVID-19 antiviral therapy.

### 2.2. SARS-CoV-2 Antiviral Candidate Biological Evaluation: Experimental Screening and Prioritization

Selected candidates were evaluated for their antiviral activity in a cell culture model of SARS-CoV-2 infection. Cytopathic effect (CPE) was determined in Vero-E6 cells, which are particularly susceptible to SARS-CoV-2 infection with a high viral load resulting in general cell death after 72 h of infection. Cell death can be readily delayed and even prevented by treatment with reference antiviral compounds and may be used to identify new antivirals [12]. Thus, antiviral activity of new drugs can be revealed by the ability of a given compound to protect the cell monolayer upon infection. To effectively quantify the antiviral potential of FDA-approved drugs identified by the multi-target virtual screening described above, we tested the 173 candidates for their ability to protect Vero-E6 cells from virus-induced cell death at a fixed concentration of 10 µM. 

Infected cell monolayer integrity was assessed by crystal violet staining 72 h after inoculation at a multiplicity of infection (MOI) of 0.001. This analysis revealed 26 compounds that prevented virus-induced cell death at 10 µM and 7 compounds that were cytotoxic at this concentration (Table S4 of the Supplementary Materials). 

Both sets of compounds were counter-screened in a dose-response experiment to determine the range of concentrations capable of protecting the cell monolayer and to confirm their antiviral potential. Only one of the cytotoxic compounds, lanatoside C, revealed antiviral activity at lower concentrations, while the other 6 cytotoxic drugs did not reveal any protective activity. Six of the primary hits (posaconazole, thiostrepton, dipyridamole, hycanthone, gefitinib and pirenpirone) could not be unequivocally confirmed as they did not confer full protection against virus-induced cytopathic effect at any of the assayed doses. Moreover, eight of the candidates (loratadine, ivermectin, terfenadine, lapatinib, carvedilol, tilorone, reserpine and amoxapine) showed a narrow therapeutic window, since they conferred protection at a unique dose. Thus, these compounds were not further characterized. Niclosamide, digoxin, penfluridol, clofazimine, cepharantine, imatinib, pimozide, metergoline, mycophenolate mofetil, lanatoside C, efloxate, ebastine and protoporphyrin IX clearly prevented SARS-CoV-2-induced cytopathic effect at more than one dose and were selected for further characterization.

### 2.3. Antiviral Activity of Selected Candidates

**Figure 3:**
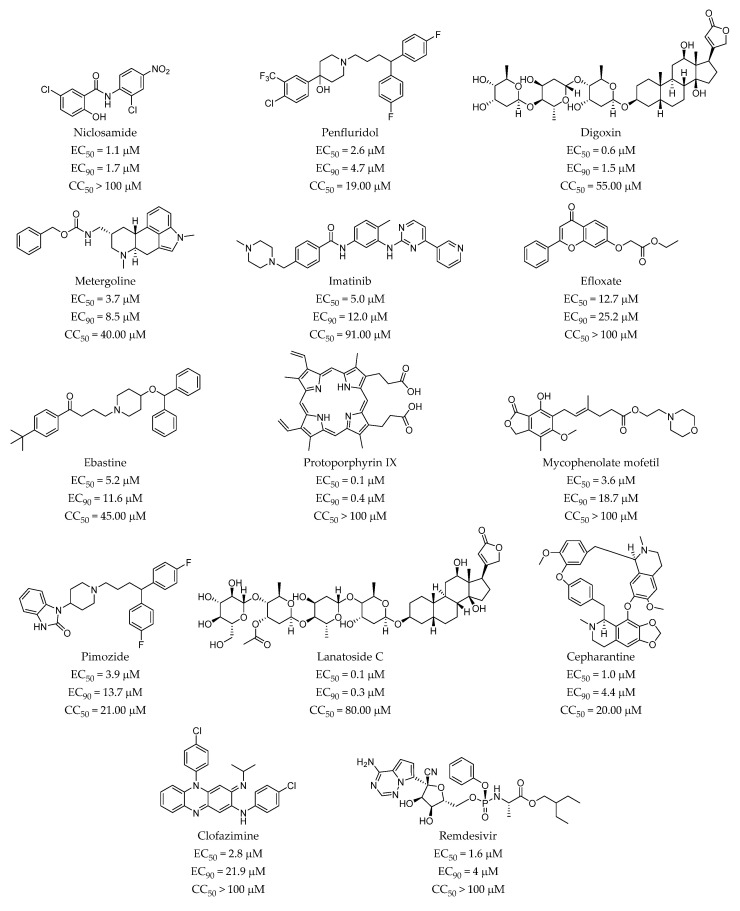
Potency and cytotoxicity indexes of the confirmed primary hits. Potency (EC_50_; EC_90_) and cytotoxicity (CC_50_) were calculated from dose-response experiments in which infection efficiency was determined by viral antigen accumulation and MTT activity respectively (see Figures S2 and S3 of the Supplementary Materials). Remdesivir was included as control of the assays.

The antiviral candidates have been selected based on their ability to prevent virus-induced cell death, which is an indirect assessment of virus infection efficiency. In order to directly confirm the antiviral activity of the selected compounds, viral antigen expression was assessed in the presence of the candidate compounds by immunofluorescence microscopy in SARS-CoV-2 infected cells using an antibody raised against SARS-CoV-2 nucleoprotein (N). Infections in the presence of a range of compound concentrations were carried out at a MOI of 0.01 and cells were fixed at 24 h post inoculation, time at which no virus-induced cytopathic effect is observed. At this time of infection and MOI, SARS-CoV-2 infection has locally spread in Vero-E6 and infection efficiency may be estimated by the expression of N protein. Staining of cell nuclei using DAPI (4,6-diamidino-2-phenylindole) allows evaluation of the cell number to verify that antiviral activity occurs at non-cytotoxic concentrations. Dose response datasets (Figure S2 of the Supplementary Materials) were used to calculate EC_50_ and EC_90_ values, corresponding to the concentration of compound that causes 50% or 90% reduction of viral antigen accumulation respectively (Figure 3). Remdesivir, a broad-spectrum nucleotide analog with anti-SARS-CoV-2 activity in vitro and in vivo was used as control and the estimated EC_50_ (1.6 µM) was similar to that previously reported in this cell line [29], indicating that the method is appropriate to estimate the potency of the compounds. 

To determine the impact of the antiviral candidates on overall cell viability, proliferation and cytotoxicity, we performed an MTT assay with a wide range of compound concentrations to estimate the CC_50_ value or the compound concentration that causes the 50% of cytotoxicity. Figure S3 of the Supplementary Materials shows the results of the MTT assay and the inferred CC_50_ values are shown in Figure 3. Based on these values, substantial antiviral activity was observed for all the compounds at non-cytotoxic doses (Figure 3). However, as it can be observed in Figure S3, treatment of the cells with niclosamide, clofazimine or protoporphyrin IX resulted in a marked, dose-dependent elevation of the MTT activity at a broad range of concentrations, suggesting that they either interfere with the assay itself or that they cause substantial stress to the cells without killing them. In the case of protoporphyrin IX and clofazimine, both compounds are colored and stain the cells significantly at the highest concentrations. While this may interfere with the interpretation of the MTT results at the highest doses, these compounds display antiviral activity at lower doses, thus reducing the concern for this phenomenon. In contrast, the lowest dose of niclosamide with antiviral activity shows substantial MTT elevation (Figures S2 and S3), probably owing to its reported mitochondrial uncoupling [30] and oxidative stress induction capacity [31]. Furthermore, elevation of the MTT activity at doses preceding marked cytotoxicity was observed in imatinib, penfluridol, pimozide, efloxate, ebastine and metergoline, probably as a transient adaptation to compound-induced stress [32]. Anyhow, and with the exception of pimozide and niclosamide, all the above-mentioned drugs showed clear antiviral activity (EC_90_) associated with cells displaying normal MTT activity (Figure S3).

### 2.4. Evaluation of Anti-SARS-CoV-2 Drugs as Entry Inhibitors

As the SARS-CoV-2 inhibitors described above were identified by a multi-target host-based entry targets screening, their ability to interfere with SARS-CoV-2 entry was evaluated in a surrogate model of infection based on retroviral vectors pseudotyped with SARS-CoV-2 Spike envelope glycoprotein. This system encompasses the production of reporter retroviral vectors pseudotyped with the envelope glycoprotein S (Spp), which is a major determinant of SARS-CoV-2 entry, mediating receptor recognition, internalization and viral membrane fusion. This system enables evaluation of virus entry efficiency as a function of the reporter gene activity (luciferase), which is strictly dependent on the presence of a functional viral glycoprotein. 

**Figure 4:**
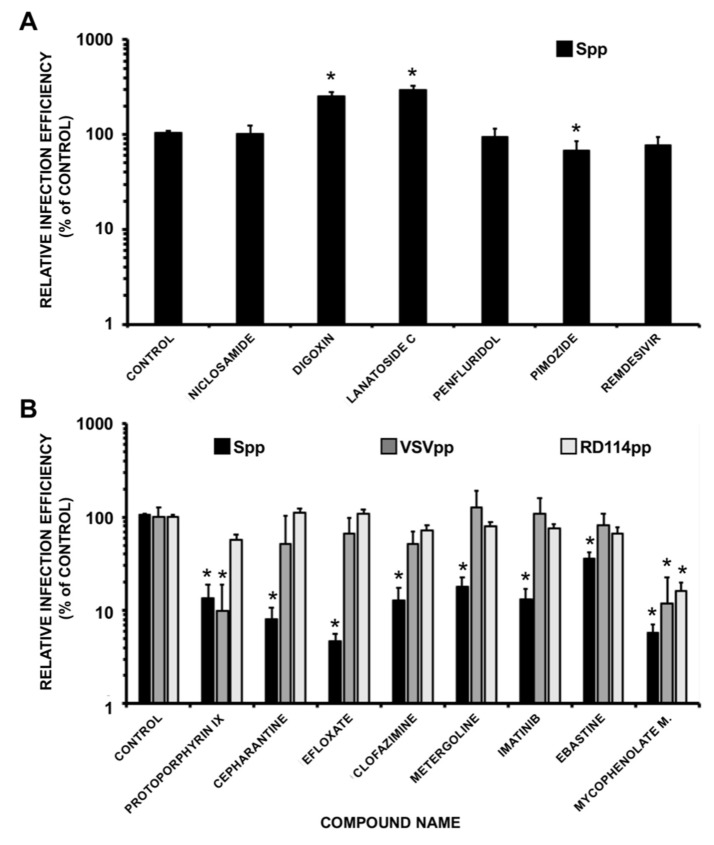
Antiviral candidates interfere with viral entry of SARS-CoV-2 pseudotypes. (**A**) Retroviral vectors pseudotyped with SARS-CoV-2 Spike glycoprotein were used to inoculate Vero-E6 cells in the presence of the candidates at the EC_90_ (see Figure 3). Forty-eight hours later, total cell lysates were assayed to determine luciferase activity as a reporter activity for viral entry. (**B**) Compound selectivity was assayed also using VSVpp and RD114pp for the compounds interfering with Spp. Data are shown as average and SEM of a minimum of four biological replicates (*N* = 4). Statistical significance was evaluated using a one-way ANOVA and a Dunnet’s post-hoc test (* *p* < 0.05).

Entry efficiency was evaluated in the presence of the EC_90_ of the candidates, except for pimozide, which was evaluated at 6.25 µM given the elevated MTT activity observed at the EC_90_ (Figure S3). This analysis revealed that, as expected, the polymerase inhibitor remdesivir did not interfere with the entry process (Figure 4A). Similarly, niclosamide, digoxin, lanatoside C, penfluridol and pimozide did not show any marked reduction in virus entry efficiency (Figure 4A) at doses capable of reducing infection efficiency by one order of magnitude (Figure 3). Thus, these results suggest that these compounds interfere with virus infection by a mechanism that does not clearly interfere with Spike-mediated entry at the assayed doses. In contrast, a significant reduction in Spp entry was observed in the presence of protoporphyrin IX, cepharantine, efloxate, clofazimine, metergoline, imatinib, mycophenolate mofetil and, although modestly, ebastine (Figure 4B). To determine if the observed inhibition is selective for SARS-CoV-2 Spike-mediated entry, pseudotypes based on vesicular stomatitis virus (VSV-G) or RD114 glycoprotein were studied in parallel. VSV-G pseudotypes use the endocytic pathway to enter the cells, although using a different receptor that S-pseudotypes and with a remarkable efficiency in many cell types [33]. On the other hand, RD114-pseudotypes, are internalized after direct fusion of the viral envelope with the cell plasma membrane and do not follow the endocytic route [34]. Mycophenolate mofetil interfered with entry of all three tested retroviral pseudotypes, probably owing to its demonstrated ability to inhibit DNA and RNA viral infections by depleting cell nucleotide pools, a function that may interfere with this retrovirus-based assay [35]. Protoporphyrin IX interfered similarly with Spp and VSVpp and to a lesser extent with RD114pp entry, suggesting non-selective interference with virus enveloped internalization, in agreement with previously reported data [36]. Partial selectivity was observed for cepharantine, efloxate, clofazimine, metergoline, imatinib and ebastine (Figure 4B).

Overall, our data suggest that interference with viral entry is a major contributor to the antiviral activity shown by cepharantine, efloxate, clofazimine, metergoline and imatinib and against SARS-CoV-2 in cell culture. 

## 3. Discussion

Since its first detection in December 2019 in Wuhan, the capital of China’s Hubei province, COVID-19 has spread worldwide rapidly. The outbreak was declared a Public Health Emergency by WHO on 30 January 2020 and since then, utmost efforts were made by the international scientific community in the attempt to find an effective cure. The full characterization of the SARS-CoV-2 viral genome by Fuk-Woo Chan J. and collaborators [37], followed by crystallization of most of its viral components offered the structural bases to search for an effective treatment. 

Vaccines represent the gold standard long-term choice to fight SARS-CoV-2 pandemic and COVID-19. However, vaccination campaigns will require a coordinated effort worldwide and full protection of the general population may be delayed for years or never be reached. Moreover, the emergence of new virus variants may reduce vaccine efficacy. Thus, pharmacological treatment of the infections with small molecules is a valid approach, but it is affected by important disadvantages, such as low potency and emergence of drug-resistant virus variants, especially when applied as monotherapy. These limitations could be dampened by the application of broad-spectrum antiviral agents simultaneously acting on more than one target at the same time [38]. Furthermore, to reduce the likelihood of resistance in future treatments, the design of antivirals able to block host targets involved in viral infection is an emerging and promising strategy [39]. In fact, this is the approach followed in this study. Thus, we screened in silico the same chemical library against eight different entry SARS-CoV-2 targets, being all of them human proteins.

The US Drug Collection of 1789 compounds of FDA-approved drugs was screened toward these targets, which consisted on the proteases TMPRSS2, Furin and Cathepsin L, the kinases AAK1, GAK and PIKfyve as well as the two-pore ion channel TPC2. Additionally, the receptor binding domain (S-RBD) of the viral Spike (S) glycoprotein, which is recognized by the host protein, ACE2 during virus attachment, was included in the analysis.

Following this trend, a hierarchical host-directed virtual screening protocol was applied to select potential anti-SARS-CoV-2 drugs based on the above-mentioned host targets with the aim to find a host-based therapy for COVID-19 capable to interfere with virus attachment, endocytosis and trafficking. In this regard, 173 FDA-approved drugs were selected from the multi-target in silico virtual screening and finally tested against SARS-CoV-2 viral infection using a high-throughput screening (HTS) protocol which was optimized for this work. 

**Figure 5:**
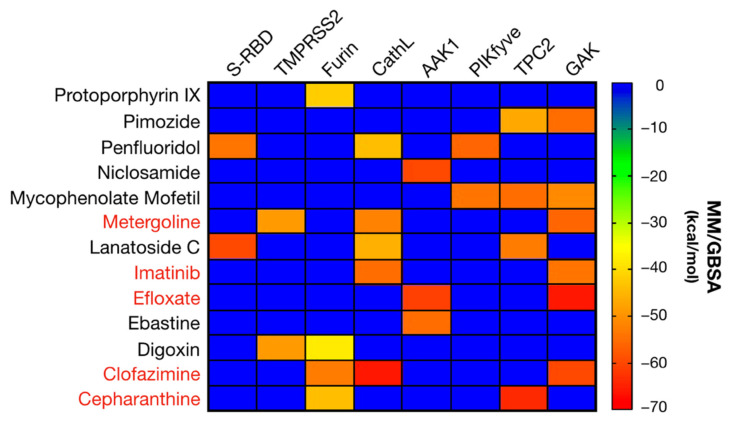
Heat matrix showing the potential targets for the 13 confirmed positive FDA compounds from Table S4 based on their MM/GBSA scores. The five finally identified as entry inhibitors are highlighted in red.

The potential antiviral activity of the selected FDA-approved drugs selected during VS was first evaluated in a cell culture model of SARS-CoV-2 infection at a fixed concentration of 10 µM. Vero-E6 cells were selected because of the proven susceptibility to the infection by this coronavirus. This preliminary assay yielded 26 hits (Table S4 of the Supplementary Materials) and subsequent dose-response experiment to determine the range of protective concentration allowed confirmation of 13 candidates with antiviral activity at non-cytotoxic doses for further studies (Figure 3). The potential targets for the selected 13 FDA-approved candidates are summarized in Figure 5. These drugs with antiviral activity virtually covering one or more targets have been selected by applying the previously described hierarchical in silico/in vitro study based on host targets involved in the viral infection.

Although in deep mechanism of action studies are needed to properly decipher the relationship between the selected drugs and the cited targets, the possibility of a multi-target antiviral effect seems to be suggested for some of them thus constituting a potentially valuable strategy to fight SARS-CoV-2 in a more effective way. In this regard, inhibition of the activity of proteases involved in spike protein degradation (furin and cathepsin L) or kinases involved in the endocytic pathway (AAK1 and GAK) is suggested to be the most plausible mechanism as evidenced by our in silico studies. Accordingly, these host-based targets could emerge as a powerful strategy for further research on anti-COVID-19 drugs.

The aim of this study was to determine if any of the antiviral candidates interferes with viral entry, as predicted by the bioinformatic analysis. A specific assay consisting on S protein pseudotyped retroviral vectors was set up to gain deeper knowledge about the potential ability of identified antivirals to inhibit SARS-CoV-2 entry. In these experiments, we confirmed that cepharantine, imatinib, efloxate, clofazimine, and metergoline prevent viral infection primarily by interfering with viral entry (Figure 4). 

**Figure 6:**
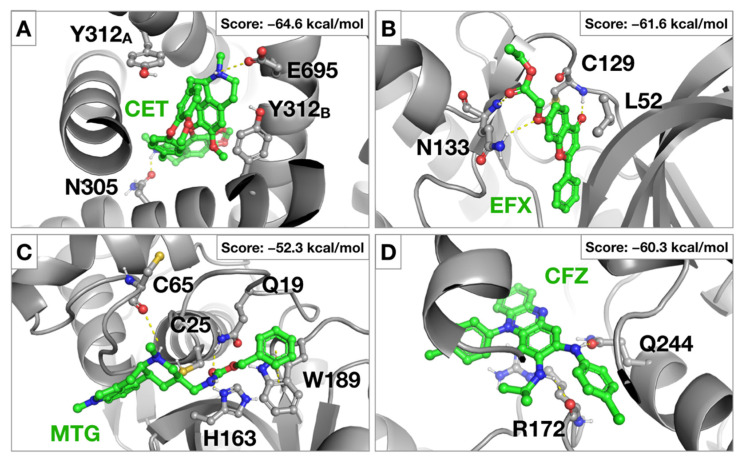
Binding modes and relative MMGBSA scores for (**A**) cepharantine (CET) in complex with TPC2, (**B**) efloxate (EFX) in complex with AAK1, (**C**) metergoline (MTG) in complex with cathepsin L and (**D**) clofazimine (CFZ) in complex with GAK.

Cepharantine was approved in Japan to treat alopecia [40], which was proposed in the last months as anti-COVID-19 therapy based on theoretical and in vitro results [11]. Our in silico results showed that cepharantine could be a potential inhibitor of furin and TPC2, being its biological action involved not only in first entry phases but also in the escape from late endosomes, a mechanism that is compatible with the results obtained in the surrogate model of viral entry here presented (Figure 4B) and a recently published report [41]. Moreover, in the last months, cepharantine has been identified as an experimental inhibitor of TPC2 [42]. The proposed binding mode for cepharantine (CET) in TPC2 is illustrated in Figure 6A. Accordingly, the compound fills the central pore of TPC2 and is stabilized by hydrophobic interactions with two residues of Y312, and hydrogen-bonding interactions with E695 and N305.

Due to the involvement of Abl pathway in viral infections, imatinib was proposed as anti-SARS-CoV-2 and clinical trials were started since the first moment of the pandemic [43], although no experimental evidence of antiviral activity was reported. At the time of writing this manuscript, in vitro activity against SARS-CoV-2 has been described [44] in agreement with the results here presented. 

To our knowledge, no antiviral activity has ever been reported for the vasodilator efloxate. Our virtual screening shows that this drug could potentially inhibit AAK1 and GAK kinases involved in early endosome entry. The proposed binding mode of efloxate (EFX) in AAK1 kinase is reported in Figure 6B. The compound is deeply inserted in the catalytic domain of AAK1 and forms hydrogen-bonding interactions with the backbone nitrogen of C129 and with Q133. 

Similarly, no antiviral activity has been reported for metergoline against SARS-CoV-2, which our VS predicts to interfere with host proteases involved in viral entry. A potential binding mode of metergoline (MTG) in the catalytic site of the protease, cathepsin L is reported in Figure 6C. The benzyl-carbamate moiety of metergoline is placed around the catalytic triad and form hydrogen-bonding interactions with H163 and W189 and π-stacking interactions with the indole moiety of W189. At the peripheral site, another hydrogen-bonding interaction involves the backbone oxygen of C75.

Clofazimine, used as an antimicrobial agent, also showed consistent antiviral activity as previously reported in a similar infection system [12]. Our data suggest that clofazimine interferes selectively with SARS-CoV-2 (Figure 4B). Its proposed binding mode in an allosteric binding site of the GAK kinase is reported in Figure 6D. Here, the protein-ligand complex is stabilized by hydrogen-bonding interactions with R172 and Q244 and a cation-π interaction formed between the central aromatic core of clofazimine (CFZ) and the guanidinium moiety of R172.

Five broad-spectrum antiviral compounds—niclosamide, digoxin, protoporphyrin IX, mycophenolate mofetil and lanatoside C—were considered in this study to determine their impact on viral entry. We confirmed antiviral activity for all five compounds. However, we did not observe interference with SARS-CoV-2 entry for lanatoside C, niclosamide or digoxin at the EC_90_, suggesting that interference with viral entry is not a major contributor to infection inhibition at the assayed concentrations. 

Consistent with our results, digoxin has been recently shown to interfere with viral replication at post-entry stages [45]. Also, lanatoside C has previously been shown to display antiviral activity against different RNA viruses at post-entry steps [46]. Finally, niclosamide displays antiviral activity against a broad range of viruses, including SARS-CoV [47] and it has been proposed that it could interfere with viral entry by preventing endosomal acidification. We failed to show this effect since we tested the compound at doses devoid of any alteration of the MTT values.

Owing to their previously described broad antiviral activity, protoporphyrin IX [36] and mycophenolate mofetil [48] displayed relatively non-specific inhibition of the retroviral pseudotyped entry and precluded concluding on their genuine impact on SARS-CoV-2 viral entry.

## 4. Materials and Methods

### 4.1. Computational Studies

#### 4.1.1. The Drug-Dataset

A starting list of 1789 FDA-approved drugs (US Drug Collection, MicroSource Discovery Systems) has been prepared for VS with the LigPrep and Epik modules of Maestro suite [49]. Accordingly, all possible ionization states at pH 7.2 ± 2.0 have been predicted for each compound. Original chirality has been retained. This led to a total of 2627 structures corresponding to the different conformers from the original library. The force field, OPLS3 [50] has been used to define all the generated compounds. 

#### 4.1.2. Protein Targets and MD Simulations 

The X-ray crystal structure of SARS-CoV-2 spike receptor binding domain in complex with ACE2 (PDB ID: 6M0J) [51] has been used as model for the S1-RBD-ACE2 recognition surface.

For TMPRSS2, the homology modelled extracellular region of the protein was obtained from the Swiss-Model repository [52]. The model was obtained from the serine protease hepsin (PDB ID: 5CE1), which shares the 34% of sequence identity with the target protein, TMPRSS2.

Amber 18 [53] was used to explore the local conformational flexibility of the S1-RBD of Spike and to refine the homology modelled structure of TMPRSS2. The ff14SB force field [54] was used to define the proteins which were embedded in a truncated octahedral TIP3P [55] water box in a layer of 22 Å and neutralized by adding chlorine counterions. Disulphide bonds were built by using the “bond” command in tleap.

Protonation states for titratable residues were set according to Propka [56] predictions at pH 7.3. Systems were energy minimized in three steps involving firstly all hydrogen atoms, then water molecules, and finally all the system. For the final step, a maximum of 50,000 (5000 iterations with steepest descent and the rest with conjugate gradient) were run. Thermalization of the minimized systems from 0 to 300 K was accomplished in five steps, the first being performed at constant volume and the rest at constant pressure. Langevin dynamics with a collision frequency of 1.0 ps^−1^ was applied for temperature regulation during thermalization. Prior to MD, 5 ns of equilibration at constant pressure were run to properly stabilize the systems. A total of 100 ns of MD production were generated in the NVT ensemble and in periodic boundary conditions for both systems. A time step of 2 fs was set for saving trajectories.

The SHAKE algorithm [57] was applied to constrain bonds involving hydrogen atoms. Cut-off for non-bonded interactions was set to 10 Å. Electrostatic interactions beyond the cut-off within the periodic box were computed by applying the Particle Mesh Ewald (PME) method [58]. The weak-coupling algorithm with a time constant of 10.0 ps was used to stabilize the temperature during the simulation. Trajectories analysis and clusterization were done by using the CPPTRAJ module of Amber18. For clustering analysis, a total of 10 clusters were preliminary searched by using the average linkage algorithm, which uses the average distance between members of two clusters [59]. Representative structure for each cluster was represented by the average structure. Cut-off for determining local density was set at 4 angstroms. Parameters for clusterization were adapted considering both trajectory and protein length.

For human PIKfyve, the structure of the protein has been obtained by homology modeling by using the crystal structure of zebrafish Phosphatidylinositol-4-phosphate 5-kinase alpha isoform with bound ATP/Ca^2+^ (PDEB code: 6CMW), which shares the 28% of global sequence identity [60]. To proper refine the ATP binding site in the homology modelled PIKfyve enzyme, ATP has been accommodated in its binding site by using the template complex as reference, and the so-derived PIKfyve-ATP complex has been then energy minimized. The ATP parameters for minimization with Amber18 were taken from the Amber parameter database of the Bryce group [61,62]. The complete list for all the crystallographic structures used during VS is reported in Table S1 of the Supplementary Materials. 

#### 4.1.3. Structure-Based Virtual Screening (SBVS) and MM-GBSA Rescoring

The post-processed FDA database of 2627 molecular candidates was screened against the previously described targets. Representative 3D-structures/clusters for S1-RBD and TMPRSS2 were selected from MD simulations. For cluster selection on S-RBD, clusters with a population higher than 5% (clusters 0-4) were considered for VS with the aim to enhance the exploration of the conformational variability of the receptor binding site of S protein. In the case of TMPRSS2, only those clusters where the active site was in an open state were considered, 3 clusters (cluster 0-2) were finally selected for VS. For both systems, the minimized structure was also considered for VS. 

For the rest of the screened systems (AAK1, Cathepsin-L, furin, GAK, PIKfyve and TPC2), the minimized crystallographic structures were prepared for virtual screening with the protein preparation wizard from the Maestro suite, applying the OPLS3e force field [50] with default parameters. The grid boxes were centered on the active site for each target (see Table S1 of the Supplementary Materials) using default parameters for receptor grid generation.

SBVS was then performed by using a pipeline which included 3 stages. The first one consisted in massive docking simulations employing the Glide software [63] and the Standard Precision (SP) method. In this first stage, an enhanced sampling approach was used, and 5 poses were generated per compound state. The best 50% of compounds (according to the scoring function) were kept and used for the second stage, where the Extra Precision (XP) method was employed. In the second stage, 25% of the best-ranked solutions were kept. Rescoring was performed in the third stage with Prime MM-GBSA method [64]. 

### 4.2. SARS-CoV-2 Infection Assays

All infection experiments were performed by inoculating Vero-E6 cells seeded onto 96-well plates (2 × 10^4^ cells/well) with the SARS-CoV-2 strain NL/2020 (kindly provided by Dr. R. Molenkamp, Erasmus University Medical Center Rotterdam) at low multiplicity of infection (MOI) of 0.01 or 0.001, as indicated below. Cultures were maintained at 37 °C in a 5% CO_2_ incubator for different lengths of time depending on the experiment. Compounds were diluted from 10 mM stock solutions in complete media containing 2% FBS to achieve the indicated final concentrations. 

#### 4.2.1. Cell Monolayer Protection Assays

For the primary screening, Vero-E6 cell monolayers were inoculated at MOI 0.001 in the presence of 10 µM of each compound in duplicate wells. Seventy-two hours later the cells were fixed and stained using crystal violet. Compounds that protect from the virus induced cell death were selected for further experiments. A wide range of two-fold dilutions of the compound (final concentration from 50 to 0.78 µM) were used in subsequent experiments to determine the maximum and minimum protective concentrations as indicated above. Only compounds conferring full protection at two consecutive dilutions (2-fold dilutions) were considered for further characterization. 

#### 4.2.2. Evaluation of the Antiviral Activity Immunofluorescence Microscopy

VeroE6 were seeded onto 96-well plates as described above and infected in the presence of the indicated compound dose (MOI 0.01). Twenty-four hours post infection, cells were fixed for 20 min at RT with a 4% formaldehyde solution in PBS, washed twice with PBS and incubated with incubation buffer (3% BSA; 0.3% Triton X100 in PBS) for 1 h. A monoclonal antibody against N protein was diluted in incubation buffer (1:2000; Genetex HL344) and incubated with the cells for 1 h, after which the cells were washed with PBS and subsequently incubated with a 1:500 dilution of a goat anti-rabbit conjugated to Alexa 488 (Invitrogen-Carlsbad, CA, USA). Nuclei were stained with DAPI (Life Technologies) during the secondary antibody incubation using the manufacturer’s recommendations. Cells were washed with PBS and imaged using an automated multimode reader (TECAN Spark Cyto; Grödig, Austria).

Clofazimine interfered with the fluorescence-based assay due to its intrinsic fluorescence [65] at the highest concentrations. Thus, this assay was performed using a colorimetric readout similar to what has previously been described [66], using a primary human monoclonal anti-S antibody (kindly provided by L. A. Fernández and J.M. Casasnovas (CNB-CSIC; Madrid, Spain) and a secondary goat anti-human Fc antibody conjugated with horseradish peroxidase.

EC_50_ and EC_90_ values were obtained using PROBIT regression method using IBM SPSS Software Package (version 26) using the average infection efficiency values from three biological replicates.

#### 4.2.3. Cytotoxicity Measurement by MTT Assays

Vero-E6 cell monolayers were treated with a wide range of compound concentrations (from 50 to 0.78 µM) and forty-eight hours later they were subjected to MTT assays using standard procedures [67]. CC_50_ values were graphically interpolated from dose-response curves obtained with three biological replicates.

### 4.3. SARS-CoV-2 Spike Protein-Pseudotyped Retroviral Vectors

Retroviral particle production pseudotyped with different viral envelopes has previously been described [68,69]. Packaging plasmids, vesicular stomatitis virus (VSV) G and RD114 glycoprotein expressing plasmids were kindly provided by Dr. F. L. Cosset (INSERM, Lyon, France). SARS-CoV-2 S expressing plasmid was obtained from Jose María Casasnovas and Juan García Arriaza (CNB-CSIC). Particles devoid of envelope glycoproteins were produced in parallel as controls.

For SARS-CoV-2 Spike pseudotyped particle (SARS2pp) entry experiments, Vero-E6 cells (10^4^ cells/well) were seeded onto 96-well plates the day before. Compounds were diluted in complete media [(DMEM supplemented with 10 mM HEPES, 1× non-essential amino acids (Gibco), 100 U/mL penicillin-streptomycin (Gibco) and 10% Fetal Bovine Serum (heat-inactivated at 56 °C for 30 min)] to achieve a 2× concentration. Fifty microliters (50 µL) of the SARS2pp, VSVpp or RD114 retrovirus dilutions were mixed 1:1 with 50 µL of the 2× compound dilutions to achieve the EC_90_. One hundred µL of the mixture was applied onto the Vero E6 cell monolayer in at least four biological replicates and cells were cultured at 37 °C in a 5% CO_2_ incubator. Twenty-four hours later, cell culture media was replaced with compound-free media. Forty-eight hours post-inoculation, cells were lysed for luciferase activity determination using Luciferase Assay System (Promega) and a luminometer. Relative infection values were determined by normalizing the data to the average relative light units detected in the vehicle control cells.

## 5. Conclusions

Overall, the use of an FDA-approved chemical library allowed us to check the robustness and reproducibility of our protocol, a multi-target virtual screening following by a solid experimental cascade of biological assays. Our study allowed for the identification and experimental validation of valuable candidates to be repurposed as potential COVID-19 therapy such as cepharantine, efloxate, metergoline, imatinib, clofazimine, digoxin, protoporphyrin IX, and lanatoside C. Moreover, a potential mechanism of action for these drugs was also proposed by in-silico VS analyses as they would be able to modulate some of the host proteins involved in the entry process of SARS-CoV-2 and was experimentally supported for cepharantine, imatinib, efloxate, clofazimine, and metergoline. In summary, we have identified a list of five drugs ready to be validated in clinical trials as SARS-CoV-2 infection inhibitors. In the case of positive results from clinical trials with COVID-19 patients, these compounds may promote a new era of antiviral agents potentially able to combat the current COVID-19 pandemic, but also future outbreaks of high pathogenic viruses, which would share a common entry pathway as an infection mechanism.

## Data Availability

The data presented in this study are available in the article and Supplementary Material and on request from the corresponding author.

## Supplementary Materials

The following are available online at https://www.mdpi.com/article/10.3390/ph14040332/s1, Figure S1: RMSD and RMSF analyses for the protein backbone atoms of S1-RBD and TMPRSS2, Figure S2: Evaluation of the antiviral candidates using immunofluorescence microscopy-based viral antigen detection, Figure S3: Evaluation of compound cytotoxicity. Table S1: Structural details about the eight targets analyzed in this study, Table S2: Clustering analysis of MD trajectories for S1-RBD and TMPRSS2, Table S3: Top ranked drugs from the FDA library by virtual screening, Table S4: Host-based target profile of compounds selected for biological evaluation on SARS-CoV-2 screen at 10 µM.
