# Supplementary material for: Host-Directed FDA-Approved Drugs with Antiviral Activity against SARS-CoV-2 Identified by Hierarchical In Silico/In Vitro Screening Methods
Source: Pharmaceuticals (Basel). 2021 Apr 6;14(4):332. doi: 10.3390/ph14040332 (PMC8067418; doi:10.3390/ph14040332)
Supplement: Supplementary file 1 [file pharmaceuticals-14-00332-s001.zip › Supplementary files_rev2/CoV-2 host-based_SuppInfo (Tables S1-S3 and Figure S1-S3).pdf]

Supporting Information

Host-directed FDA-approved drugs with antiviral activity against SARS-CoV-2 identified by hierarchical *in silico/in vitro* screening methods

Tiziana Ginex,[1, §] Urtzi Garaigorta,[2, §] David Ramírez,[3] Victoria Castro,[2] Vanesa Nozal,[1] Ines Maestro,[1] Javier García-Cárceles,[1] Nuria E. Campillo,[1] Ana Martinez,[1] Pablo Gastaminza,[2,*] Carmen Gil[1,*]

[1]Centro de Investigaciones Biológicas Margarita Salas-CSIC, Ramiro de Maeztu 9, 28040 Madrid (Spain)

[2]Centro Nacional de Biotecnologia-CSIC, Calle Darwin 3, 28049 Madrid (Spain)

[3]Instituto de Ciencias Biomédicas, Universidad Autónoma de Chile, Llano Subercaseaux 2801 – piso 6, Santiago, Chile.

[§]Both authors contribute equally to this work

Index

Page S2: Table S1 Structural details about the eight targets analyzed in this study.

Page S3: Table S2 Clustering analysis of MD trajectories for S1-RBD and TMPRSS2.

Page S4: Top ranked drugs from the FDA Library by Virtual screening: MMGBSA values and controls used for each target studied.

Page S14: Figure S1 RMSD and RMSF analyses for the protein backbone atoms of S1-RBD and TMPRSS2.

Page S15: Figure S2 Evaluation of the antiviral candidates using immunofluorescence microscopy-based viral antigen detection,

Page S16: Figure S3 Evaluation of compound cytotoxicity.

Table S1. Structural details about the eight targets analyzed in this study.

Targets	Available PDB structures[a]	Selected PDB structure	Number of conformations used for VS	Binding sites	Control inhibitors
**Virus-host**					
**S1-RBD**	301	6M0J	6<br>- 1 Minimized<br>- 5 Clusters (0-4) from MD	**Active site:**<br>F154, Y157, N155, K85, N161, Y117, Y121, Q166, T168, N169, Y173, Y173	**-**
**Host**					
**TMPRSS2**	-	Homology modeling from 5CE1 (TMPRSS)	4<br>- 1 Minimized<br>- 3 Clusters (0-2) from MD	**Active site:**<br>S296, T314, D290, S291, V135, W163, C136 C152	camostat<br>nafamostat<br>bromhexine
**AAK-1**	3	4WSQ	1<br>- Minimized	**Active site:**<br>G55, K74, M126, D127, F128, Q133, L183, D194	sunitinib<br>baricitinib<br>5L4Q-ligand (**LKB**)
			1<br>- Minimized	**Allosteric site:**<br>H82, R89, R175, D176, S197, A198, T199, N200, Q203, E208, A212, E216	gefitinib
**Cathepsin-L**	38	4AXL	1<br>- Minimized	**Active site:**<br>C25, H163, N187	oxocarbazate
**Furin**	21	5MIM	1<br>- Minimized	**Active site:**<br>H194, N295, S368.	5MIM-ligand (**1n**)
**GAK**	7	5Y7Z<br>(Active Site)	1<br>- Minimized	**Active site:**<br>K69, E124, C126, Q129, D191	Wee1/Chk1<br>gefitinib<br>bosutinib
		5Y80<br>(Allosteric site)	1<br>- Minimized	**Allosteric site:**<br>D173, H200, T223, S194, N221	gefitinib
**PIKfyve**	-	Homology Modeling from 6CMW (zebrafish PIP5K)	1<br>- Minimized	**Active site:**<br>G19, K20, S21, A23, F25, I34, K36, 96, E97, N98, L99, F100, D110, K112, V132, L179, I194, D195, R198	apilimod<br>YM201636
**TPC2**	3	6NQ0 (Open)<br>6NQ2 (Closed)	2<br>- Minimized open<br>- Minimized closed	**Main site:**<br>N305, A691	raloxifen<br>tamoxifen<br>pimozide<br>tetrandrine<br>fluphenazine<br>verapamil-S
			2<br>- Minimized open<br>- Minimized closed	**Secondary site:**<br>W157, R210, F193	ned19

[a]Last accession: October 20, 2020. Only structures for *Homo sapiens* for host-based targets were considered.

Table S2. Clustering analysis of MD trajectories for S1-RBD and TMPRSS2.

	S1-RBD		TMPRSS2	
**# Clusters***	Population	Frac	Population	Frac
0	1721	43.0%	625	16.1%
1	739	18.5%	606	15.6%
2	595	14.9%	561	14.4%
3	452	11.3%	406	10.4%**
4	354	8.8%	395	10.2%**
5	62	1.5%***	367	9.4%**
6	48	1.2%***	333	8.6%**
7	26	0.6%***	329	8.5%**
8	2	0.1%***	149	3.8%**
9	1	<0.1%***	115	3.0%**

* A total of 10 clusters were preliminary searched. Cut-off for determining local density was 4 angstroms. Average linkage algorithm, which uses the average distance between members of two clusters, was applied.

** Discarded because the catalytic site is closed.

*** Discarded since population is less than 5%.

Table S3. Top ranked drugs from the FDA Library by Virtual screening: MMGBSA values and controls used for each target studied.

ACE2-S

No	Drug name	MMGBSA (Kcal.mol^{-1})	
1	PENTAGASTRIN	-75.578	
2	RITONAVIR	-72.177	
3	NONOXYNOL-9	-70.87	Not selected. Cosmetic ingredient
4	BENZONATATE	-68.252	
5	LINACLOTIDE	-68.036	
6	NADIDE	-67.116	
7	TILMICOSIN	-65.933	Not selected. Veterinary drug
8	THIOSTREPTON	-64.685	
9	DIRITHROMYCIN	-61.916	
10	CANDICIDIN	-60.409	Not selected. Vaginal administration
11	LANATOSIDE C	-60.163	
12	ETOPOSIDE	-58.7	
13	AMPHOTERICIN B	-58.473	
14	beta-CAROTENE	-58.21	
15	IOXILAN	-58.045	Not selected. Contrast agent
16	ESCIN	-57.95	
17	DACTINOMYCIN	-57.789	
18	CANAGLIFLOZIN	-57.618	
19	PANTETHINE	-57.003	
20	DIGITOXIN	-56.284	
21	CEFOPERAZONE	-56.216	
22	ACARBOSE	-56.138	
23	SILIBININE	-56.054	
24	IVERMECTIN	-55.217	
25	TOLTRAZURIL	-55.053	Not selected. Veterinary drug
26	SENNOSIDE A	-54.841	Not selected. Strong laxative
27	SUVOREXANT	-54.778	
28	PENFLURIDOL	-54.723	
29	CEFDITORIN PIVOXIL	-54.694	
30	SPIRAMYCIN	-53.601	
31	PROSCILLARIDIN A	-53.579	
32	ADEFOVIR DIPIVOXYL	-53.423	
33	TERFENADINE	-53.219	
34	TELITHROMYCIN	-53.218	
35	CEFPIRAMIDE	-53.212	
36	TENIPOSIDE	-53.095	
37	NYSTATIN	-53	
38	MUPIROCIN	-52.942	
39	LACTULOSE	-52.927	
40	RIFAXIMIN	-52.887	
41	INDINAVIR SULFATE	-52.783	
42	BISOCTRIZOLE	-52.684	
43	CEPHALONIUM	-52.662	
44	KITASAMYCINS	-52.638	
45	RUTIN	-52.577	
46	DIOSMIN	-52.142	
47	RACECADOTRIL	-51.857	
48	TROXERUTIN	-51.8	
49	DROPERIDOL	-51.743	
50	LOPINAVIR	-50.994	

No reference compounds were used. These are the 50 top ranked compounds

TMPRSS2

No	Drug name	MMGBSA (Kcal.mol^{-1})	
1	NONOXYNOL-9	-78.098	Not selected. Cosmetic ingredient
2	TILMICOSIN	-62.126	
3	INDINAVIR SULFATE	-60.346	
4	LAPATINIB	-59.217	
5	SENNOSIDE A	-57.548	Not selected. Strong laxative
6	TELITHROMYCIN	-57.161	
7	DOXORUBICIN	-57.021	
8	PACLITAXEL	-57.008	
9	CANAGLIFLOZIN	56.252	
10	NADIDE	-55.618	
11	PANTETHINE	55.469	
12	CHLORAMPHENICOL PALMITATE	-55.232	
13	ALOIN	-55.017	
14	DIOSMIN	-54.968	
15	CASANTHRANOL	-54.683	Not selected. Strong laxative
16	DIPYRIDAMOLE	-54.325	
17	AMPHOTERICIN B	-53.671	
18	CEFPODOXIME PROXETIL	-52.074	
19	RIFAXIMIN	-52.035	
20	TELMISARTAN	-51.88	
21	ACARBOSE	-51.721	
22	RITONAVIR	-51.71	
23	PREDNISOLONE ACETATE	-51.709	
24	MUPIROCIN	-51.669	
25	ERYTHROMYCIN ETHYLSUCCINATE	-50.953	
26	SUCRALOSE	-50.881	
27	TOBRAMYCIN	-50.208	
28	EMPAGLIFLOZIN	-50.196	
29	SILIBININ	-50.022	
30	DI-O-DEMETHYLCURCUMIN	-49.817	
31	DIGOXIN	-49.647	
32	CAPECITABINE	-49.287	
33	DORAMECTIN		
34	FOLIC ACID	-49.108	
35	PENTAGASTRIN	-49.002	
36	METERGOLINE	-48.979	
37	PYRITINOL	-48.761	
38	DIGITOXIN	-48.602	
39	DIBEKACIN	-48.31	
40	GLAFENINE	-48.163	
41	CIANIDANOL	-48.154	
42	KITASAMYCINS	-47.972	
43	IOVERSOL	-47.408	Not selected. Contrast agent
44	PROTIRELIN	-47.204	
45	TERFENADINE	-47.081	
46	ZOLPIDEM	-46.875	
47	CEFPIRAMIDE	-46.861	
48	COLCHICINE	-46.397	
49	CHLORALOSE	-46.037	Not selected. Raticide
**Reference compounds**			
	CAMOSTAT	-46.151	
	NAFAMOSTAT	-44.812	

	BROMHEXINE	-31.454

Furin

No	Drug name	MMGBSA (Kcal.mol^{-1})	
1	DIBEKACIN	-71.612	
2	ACARBOSE	-58.223	
3	KETOCONAZOLE	-57.905	
4	PROTIRELIN	-56.713	
5	CLOFAZIMINE	-53.168	
6	AZITHROMYCIN	-52.296	
7	TELITHROMYCIN	-52.04	
8	TOBRAMYCIN	-51.705	
9	DOXORUBICIN	-51.231	
10	OLMESARTAN MEDOXOMIL	-51.167	
11	TERCONAZOLE	-49.62	Not selected. Vaginal administration
12	TILMICOSIN	-49.094	
13	TIOCONAZOLE	-48.643	Not selected. Topical use
14	CANDICIDIN	-48.522	Not selected. Vaginal administration
15	AZACITIDINE	-48.399	
16	TILORONE	-48.098	
17	RESERPINE	-47.906	
18	KETANSERIN	-47.802	
19	LEVOFLOXACIN	-46.515	
20	ATENOLOL	-45.586	
21	FAMOTIDINE	-45.569	
22	RUTIN	-45.447	
23	OLANZAPINE	-45.362	
24	DIOSMIN	-45.245	
25	HYCANTHONE	-45.143	
26	AMSACRINE	-44.555	
27	CEPHARANTHINE	-44.369	
28	PERPHENAZINE	-43.941	
29	PANTOPRAZOLE	-43.448	
30	OMEPRAZOLE	-43.283	
31	PROTOPORPHYRIN IX	-42.845	
32	RIFAXIMIN	-42.751	
33	DIGITOXIN	-42.52	
34	CEFDITORIN PIVOXIL	-42.11	
35	BIFONAZOLE	-41.284	
36	LINAGLIPTIN	-40.729	
37	NAFTOPIDIL	-40.708	
38	ZOLPIDEM	-40.707	
39	GLAFENINE	-40.643	
40	ITOPRIDE HYDROCHLORIDE	-40.488	
41	METAPROTERENOL	-40.247	
42	SIROLIMUS	-40.18	
43	TRIMETHOPRIM	-39.921	
44	LUMEFANTRINE	-39.636	
45	BRIMONIDINE	-39.552	Not selected. Oftalmic drops
46	DIAVERIDINE	-39.453	Not selected. Veterinary drug
47	PALIPERIDONE	-39.377	
48	SPIRAMYCIN	-39.299	
49	PIRENPERONE	-39.233	
50	CEFPODOXIME PROXETIL	-39.174	
51	CASANTHRANOL	-39.037	Not selected. Strong laxative
52	CEFUROXIME AXETIL	-38.846	

53	AMOXAPINE	-38.785
54	BRINZOLAMIDE	-38.742
55	MIGLITOL	-38.268
56	INDINAVIR SULFATE	-38.133
57	DIGOXIN	-38.063
58	EPROBEMIDE	-37.614
59	DAPAGLIFLOZIN	-37.544
60	AJMALINE	-37.38
61	DIRITHROMYCIN	-37.305
62	GEFITINIB	-37.088
63	MANIDIPINE HYDROCHLORIDE	-37.078
64	THIAMPHENICOL	-37.032
**Reference compounds**		
	5MIM_ligand1	-71.53
	CompoundDC1	-37.908
	CompoundB1	-36.758

Cathepsin L

No	Drug name	MMGBSA (Kcal.mol^{-1})	
1	DIRITHROMYCIN	-72.14	
2	CLOFAZIMINE	-65.981	
3	PACLITAXEL	-62.962	
4	TELITHROMYCIN	-61.943	
5	ELLAGIC ACID	-61.746	
6	EMPAGLIFLOZIN	-61.511	
7	GLAFENINE	-58.667	
8	ORBIFLOXACIN	-58.113	Not selected. Veterinary drug
9	KETOCONAZOLE	-57.356	
10	RESERPINE	-57.307	
11	ZOLPIDEM	-56.429	
12	TOBRAMYCIN	-55.297	
13	SPARFLOXACIN	-55.228	
14	IMATINIB	-54.975	
15	CLIMBAZOLE	-54.814	
16	RUTIN	-54.482	
17	NADOLOL	-54.15	
19	NAFTOPIDIL	-54.025	
20	ALOIN	-53.31	
21	TELMISARTAN	-52.716	
22	LAPATINIB	-52.512	
23	OXETHAZAINE	-52.477	
24	METERGOLINE	-52.324	
25	RIBOFLAVIN	-52.306	
26	ACARBOSE	-52.196	
27	ADENOSINE	-50.892	
28	DAPAGLIFLOZIN	-50.457	
29	EZETIMIBE	-50.372	
30	INDINAVIR SULFATE	-49.704	
31	CIANIDANOL	-49.664	
32	BROMPERIDOL	-49.214	
33	CEFALONIUM	-49.123	
34	CEFPODOXIME PROXETIL	-48.015	
35	CARVEDILOL	-47.542	
36	PANTOPRAZOLE	-47.324	
37	CILOSTAZOL	-47.044	
38	CASANTHRANOL	-47.025	Not selected. Strong laxative

39	CAPECITABINE	-46.918
40	BRIVUDINE	-46.918
41	HEXESTROL	-46.484
42	LANATOSIDE C	-46.443
43	OMEPRAZOLE	-46.063
44	CABAZITAXEL	-46.043
45	TIGECYCLINE	-45.701
46	CHLORAMPHENICOL	-45.367
47	AZITHROMYCIN	-45.002
48	SILIBININ	-44.898
49	PENTAGASTRIN	-44.417
50	PIRENPERONE	-44.415
51	BERGENIN	-44.243
52	PENFLURIDOL	-44.146
**Reference compounds**		
	Ligand_2YJC1	-60.3
	3bc3_ligand	-55.817
	Propeptidimimetic	-55.248
	Oxocarbazate	-40.762
	SID2661509	-40.449
	azapeptide	-40.181

AAK1

No	Drug name	MMGBSA (Kcal.mol^{-1})	
1	BENZONATATE	-72.657	
2	DIOSMIN	-69.592	
3	NONOXYNOL-9	-66.803	Not selected. Cosmetic ingredient
4	PERPHENAZINE	-66.461	
5	PYRITINOL	-66.029	
6	EMPAGLIFLOZIN	-61.804	
7	EFLOXATE	-61.579	
8	GLAFENINE	-60.817	
9	NICLOSAMIDE	-60.742	
10	TROXERUTIN	-60.405	
11	LAPATINIB	-59.137	
12	DAPAGLIFLOZIN	-58.52	
13	ADEFOVIR DIPIVOXYL	-58.329	
14	DANTHRON	-57.872	Not selected. Strong laxative
16	DI-O-DEMETHYL-CURCUMIN	57.714	
17	HYCANTHONE	-57.56	
18	ELLAGIC ACID	-56.911	
19	DYPHYLLINE	-53.646	
20	ANTHRALIN	-56.435	
21	ACETAMINOSALOL	-55.437	
22	COUMOPHOS	-55.095	Not selected. Insecticide
23	TELITHROMYCIN	-55.069	
24	EBASTINE	-54.99	
25	OFLOXACIN	-54.957	
26	CEFUROXIME AXETIL	-53.754	

27	PHYTONADIONE [5mM]	-53.431	
28	ESTRADIOL BENZOATE	-53.41	
29	INDOPROFEN	-52.359	
30	NOCODAZOLE	-52.327	
31	CIANIDANOL	-51.355	
32	PANTETHINE	-51.281	
33	TEPOXALIN	-51.119	Not selected. Veterinary drug
34	DOXORUBICIN	-51.026	
35	GEFITINIB	-50.316	
36	DICHLOROPHEN	-50.107	Not selected. Veterinary drug
37	IDEBENONE	-50.102	
38	alpha-TOCHOPHEROL	-50.094	
39	NORFLOXACIN	-49.778	
40	CANAGLIFLOZIN	-49.778	
41	ENOXACIN	-49.773	
42	CEFALONIUM	-49.429	
43	BERGENIN	-49.175	
44	NADOLOL	-48.984	
**Reference compounds**			
	4WSQ_ligand	-67.88	
	5TEO_LIG	-62.234	
	Baricitinib	-57.867	
	5L4Q_lig	-57.135	
	Gefitinib	-34.972	

PIKfyve

No	Drug name	MMGBSA (Kcal.mol^{-1})	
1	RITONAVIR	-98.136	
2	NONOXYNOL-9	-76.417	Not selected. Cosmetic ingredient
3	KITASAMYCINS	-74.968	
4	CHLORAMPHENICOL PALMITATE	-68.991	
5	OLMESARTAN MEDOXOMIL	-65.743	
6	TEPOXALIN	-65.153	Not selected. Veterinary drug
7	ACARBOSE	-64.351	
8	LOPINAVIR	-64.272	
9	CEFUROXIME AXETIL	-61.211	
10	PODOFILOX	-61.143	Not selected. Topic application
11	TIOCONAZOLE	-61.122	Not selected. Topic application
12	NAFTOPIDIL	-60.156	
13	CASANTHRANOL	-58.984	Not selected. Strong laxative
14	PREDNISOLONE HEMISUCCINATE	-57.044	
15	PENFLURIDOL	-56.614	
16	AMCINONIDE	-55.285	Not selected. Topic application
17	CLOTRIMAZOLE	-55.216	
18	AVANAFIL	-55.175	
19	CEFOPERAZONE	-55.096	

20	GEFITINIB	-54.89	
21	PHENOLPHTHALEIN	-54.823	Not selected. pH indicator
22	MYCOPHENOLATE MOFETIL	-54.712	
23	DI-O-DEMETHYL-CURCUMIN	-54.641	
24	HYDROCORTISONE HEMISUCCINATE	-54.228	
25	ASCORBYL PALMITATE	-54.204	
26	DERACOXIB	-54.1	Not selected. Veterinary drug
27	DOXORUBICIN	-54.10	
28	SERATRODAST	-53.996	
29	CANAGLIFLOZIN	-53.949	
30	OCTOCRYLENE	-53.915	Not selected. Cosmetic sun filter
31	OXETHAZAINE	-53.692	Not selected. Potent anestesic
32	NEFAZODONE HYDROCHLORIDE	-53.604	
33	AZAPERONE	-53.598	Not selected. Veterinary drug
34	ACEMETACIN	-53.534	
35	DROPERIDOL	-53.212	
36	TERFENADINE	-53.19	
37	LORATADINE	-52.593	
38	FENOFIBRIC ACID	-52.204	
39	LAPATINIB	-52.154	
40	CLIMBAZOLE	-52.135	Not selected. Topic application
41	CABAZITAXEL	-52.118	
42	INDINAVIR SULFATE	-51.812	
43	CAPSAICIN	-51.775	Not selected. Topic application
44	GLAFENINE	-51.65	
45	RIBOFLAVIN	-51.482	
46	PROSCILLARIDIN A	-51.288	
47	CELECOXIB	-51.138	
48	PIRIBEDIL HYDROCHLORIDE	-51.126	
49	AVOBENZONE	-51.126	Not selected. Cosmetic sun filter
50	SULFASALAZINE	-51.009	
51	RACECADOTRIL	-50.938	
52	CARVEDILOL	-50.937	
53	PHENOLSULFON-PHTHALEIN	-50.799	Not selected. pH indicator
54	MEBENDAZOLE	-50.739	
**Reference compounds**			
	Apilimod	-52,92	
	YM201636	-50,739	

TPC2

No	Drug name	MMGBSA (Kcal.mol^{-1})	
1	CASANTHRANOL	-81.125	Not selected. Strong laxative
2	TROXERUTIN	-80.572	
3	POSACONAZOLE	-78.636	
4	CANDICIDIN	-75.041	Not selected. Topical use
5	BEMOTRIZINOL	-73.155	Not selected. Cosmetic sun screen
6	CEFPODOXIME PROXETIL	-70.488	
7	CEFOPERAZONE	-66.904	
8	TELMISARTAN	-65.780	

9	CEPHARANTHINE	-64.637	
10	TILMICOSIN	-63.858	
11	CEFDITORIN PIVOXIL	-63.377	
12	ESTRIOL TRIPROPIONATE	-63.146	
13	DIOSMIN	-62.722	
14	DIPYRIDAMOLE	-62.654	
15	TELITHROMYCIN	-62.397	
16	LOPINAVIR	-62.244	
17	GLYCYRRHIZIN	-61.566	
18	TENIPOSIDE	-60.842	
19	CANDESARTAN CILEXTIL	-60.793	
20	NADIDE	-60.028	
21	ACARBOSE	-60.001	
22	OLMESARTAN MEDOXOMIL	-59.428	
23	BETAMETHASONE VALERATE	-57.152	
24	RIFAPENTINE	-56.670	
25	INDINAVIR SULFATE	-56.655	
26	MUPIROCIN	-56.513	
27	CAPECITABINE	-55.893	
28	ACEMETACIN	-55.859	
29	MYCOPHENOLATE MOFETIL	-55.703	
30	PREDNISOLONE HEMISUCCINATE	-55.588	
31	VOGLIBOSE	-54.901	
32	RAMIPRIL	-54.599	
33	CANAGLIFLOZIN	-54.081	
34	DESONIDE	-53.949	
35	LANATOSIDE C	-53.290	
36	AMCINONIDE	-53.246	Not selected.Topical use
37	PRULIFLOXACIN	-53.173	
38	ETOPOSIDE	-52.985	
39	TRIAMCINOLONE ACETONIDE	-52.889	
40	TIGECYCLINE	-52.883	
41	OXIGLUTATIONE	-52.572	
42	TEPOXALIN	-52.242	Not selected. Veterinary drug
43	CHENODIOL	-52.220	Not selected. Hepatotoxic
44	URSODIOL	-52.220	Not selected. Hepatotoxic
45	RESERPINE	-51.907	
**Reference compounds**			
	Raloxifene	-66.306	
	Tamoxifen	-55.876	
	Verapamil-S	-53.397	
	Clomiphene	-49.982	
	Fluphenazin	-48.135	
	Tetrandine	-50.288	
	Bisbenzylisoquinoline	-47.631	
	Pimozide	-47.467	
	Ned-19	-43.640	

GAK

No	Drug name	MMGBSA (Kcal.mol^{-1})	
1	GLAFENINE	-69.481	
2	LAPATINIB	-66.346	
3	TILORONE	-66.265	
4	EFLOXATE	-66.039	
5	SILIBININ	-64.614	
6	GEFITINIB	-62.580	
7	MUPIROCIN	-61.684	
8	CANAGLIFLOZIN	-61.433	
9	CLOFAZIMINE	-60.275	
10	TROXERUTIN	-59.985	
11	DECOQUINATE	-59.790	
12	BENZOYL PEROXIDE	-59.479	Not selected. Topical use
13	RETINOL	-59.198	Not selected. Topical use
14	NOCODAZOLE	-58.939	
15	PIMOBENDAN	-58.795	Not selected. Veterinary drug
16	MEBENDAZOLE	-58.391	
17	METERGOLINE	-56.191	
18	CARVEDILOL	-55.942	
19	QUINOXYFEN	-55.941	Not selected. Veterinary use
20	FLUBENDAZOLE	-55.940	
21	PIMOZIDE	-55.820	
23	BERGENIN	-55.319	
24	DIACERIN	-55.220	
25	PRULIFLOXACIN	-55.025	
26	TELMISARTAN	-54.778	
27	PERPHENAZINE	-54.182	
28	IMATINIB	-54.161	
29	TEPOXALIN	-53.937	Not selected. Veterinary drug
30	RIBOFLAVIN	-53.770	
31	AVOBENZONE	-53.170	Not selected. Cosmetic sun screen
32	FENOFIBRATE	-52.902	
33	OXFENDAZOLE	-52.871	
34	DOXORUBICIN	-52.846	
35	DERACOXIB	-52.797	
36	IPRIFLAVONE	-52.475	
37	BROMOPRIDE	-52.077	
38	KETOCONAZOLE	-52.064	
39	ROTENONE	-51.995	Not selected. Insecticide
40	NADIDE	-51.806	
41	GENISTEIN	-51.706	
42	ELLAGIC ACID	-51.448	
43	OCTINOXATE	-51.349	Not selected. Cosmetic sun screen
44	TIGECYCLINE	-51.315	
45	CEFOPERAZONE	-51.247	

46	MYCOPHENOLATE MOFETIL	-51.186	
47	CELECOXIB	-50.638	
48	AVANAFIL	-50.542	
49	TIOCONAZOLE	-50.495	Not selected. Topical use
50	PODOFILOX	-50.456	Not selected. Topical use
51	NAFTOPIDIL	-50.410	
52	CAPSAICIN	-50.266	Not selected. Topical use
**Reference compounds (catalytic)**			
	indirubinE804	-67.322	
	Wee1/ChK1	-54.502	
	Gefitinib	-58.537	
	Bosutinib	-59.341	
	Wee1/Chk1	-38.445	
**Reference compounds (allosteric)**			
	indirrubinE804	-56.831	
	Bosutinib	-46.079	
	Gefitinib	-59.014	
	GAKSknapp	-38.112	

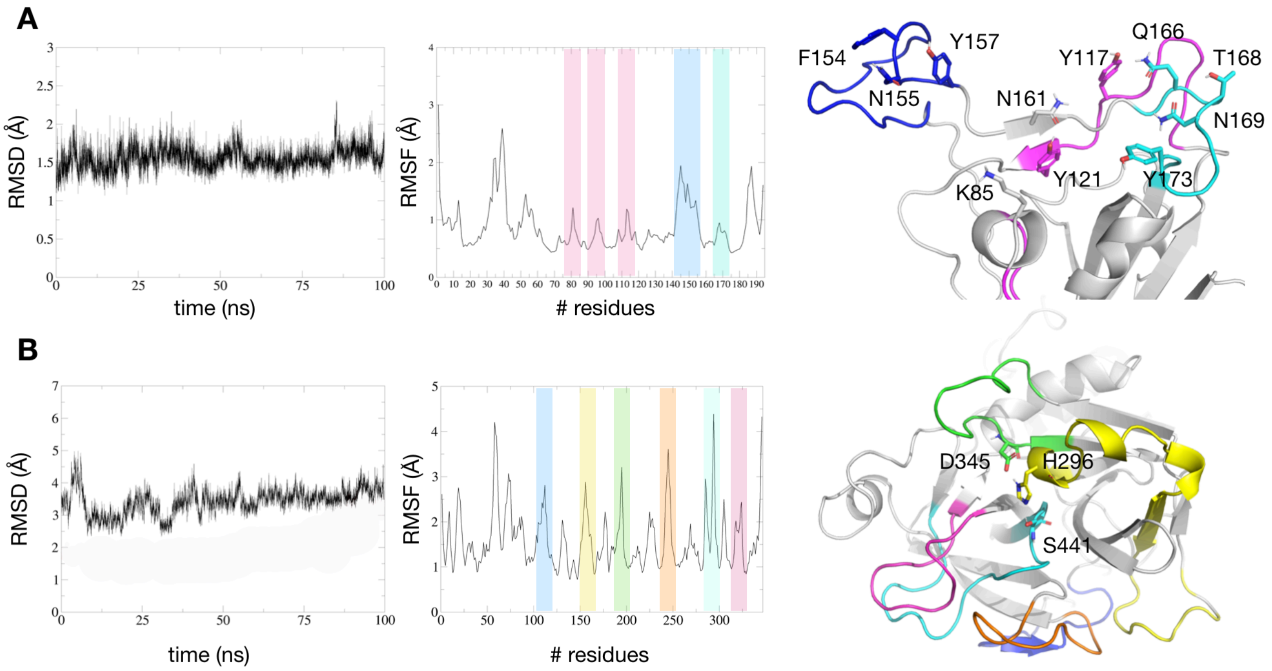

Figure S1. RMSD and RMSF analyses for the protein backbone atoms of (**A**) S1-RBD and (**B**) TMPRSS2. The energy minimized structure of the proteins has been used as reference for MD trajectory alignment, prior to RMSD and RMSF analyses. For both proteins, residues mainly involved in protein-ligand interactions are reported in sticks. Colored bands in the RMSF plots identify relevant regions pertaining to the binding sites.

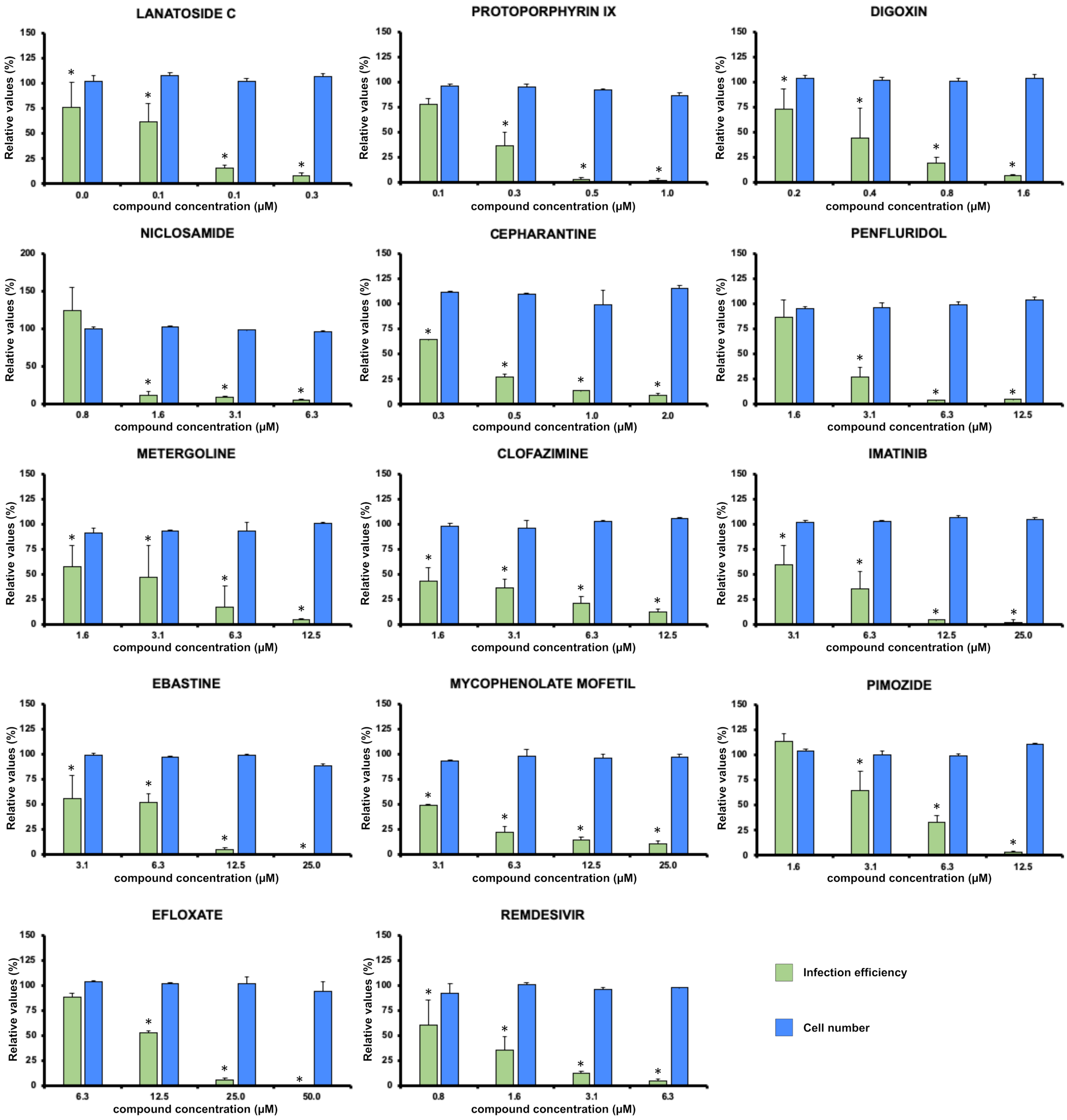

Figure S2. Evaluation of the antiviral candidates using immunofluorescence microscopy-based viral antigen detection. Vero-E6 cells were inoculated at MOI 0.01 in the presence of increasing compound concentrations. Relative infection efficiency was determined 24 hours post-infection by automated segmentation and signal quantitation using mock-infected cells and vehicle-treated cells as controls. Data are shown as average and standard deviation of three biological replicates. Statistical significance was determined using one-way ANOVA and Dunnet´s post-hoc test.

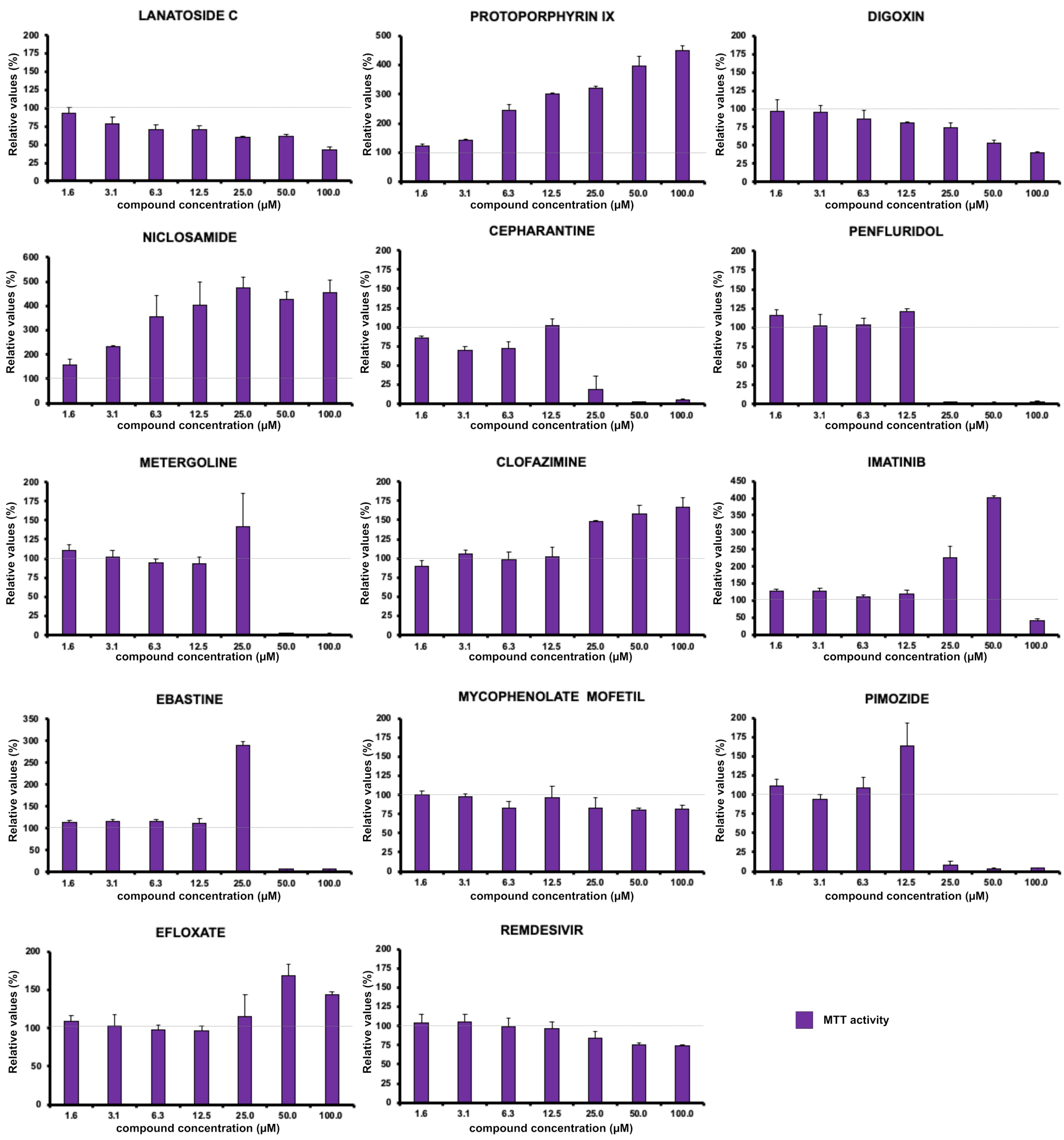

Figure S3. Evaluation of compound cytotoxicity. Vero-E6 cells were cultured in the presence of increasing compound concentrations. MTT activity was determined 48 hours post-treatment. Data are shown as average and standard deviation of three biological replicates.
